# Transcriptional and Posttranslational Regulation of Nucleotide Excision Repair: The Guardian of the Genome against Ultraviolet Radiation

**DOI:** 10.3390/ijms17111840

**Published:** 2016-11-04

**Authors:** Jeong-Min Park, Tae-Hong Kang

**Affiliations:** Department of Biological Science, Dong-A University, Busan 49315, Korea; zmpark@donga.ac.kr

**Keywords:** ultraviolet radiation, nucleotide excision repair, transcriptional regulation, posttranslational regulation

## Abstract

Ultraviolet (UV) radiation from sunlight represents a constant threat to genome stability by generating modified DNA bases such as cyclobutane pyrimidine dimers (CPD) and pyrimidine-pyrimidone (6-4) photoproducts (6-4PP). If unrepaired, these lesions can have deleterious effects, including skin cancer. Mammalian cells are able to neutralize UV-induced photolesions through nucleotide excision repair (NER). The NER pathway has multiple components including seven xeroderma pigmentosum (XP) proteins (XPA to XPG) and numerous auxiliary factors, including ataxia telangiectasia and Rad3-related (ATR) protein kinase and RCC1 like domain (RLD) and homologous to the E6-AP carboxyl terminus (HECT) domain containing E3 ubiquitin protein ligase 2 (HERC2). In this review we highlight recent data on the transcriptional and posttranslational regulation of NER activity.

## 1. Introduction

Ultraviolet (UV) radiation from sunlight continuously damages an organism′s genome. The solar UV spectrum can be divided into UVA (315–400 nm), UVB (280–315 nm), and UVC (100–280 nm) radiation [1,2]. Short-wavelength UVC (especially 254 nm) and medium-wavelength UVB readily produce DNA photoproducts such as cyclobutane pyrimidine dimers (CPD) and pyrimidine-pyrimidone (6-4) photoproducts (6-4PP) [3,4]. Because these photolesions interfere with DNA replication and transcription, they can lead to cell death and mutagenesis, thereby threatening genome stability [5]. Furthermore, these lesions can induce UV signature mutations (C → T transition and CC → TT tandem double mutations), which are the main cause of photocarcinogenesis [6,7].

**Figure 1:**
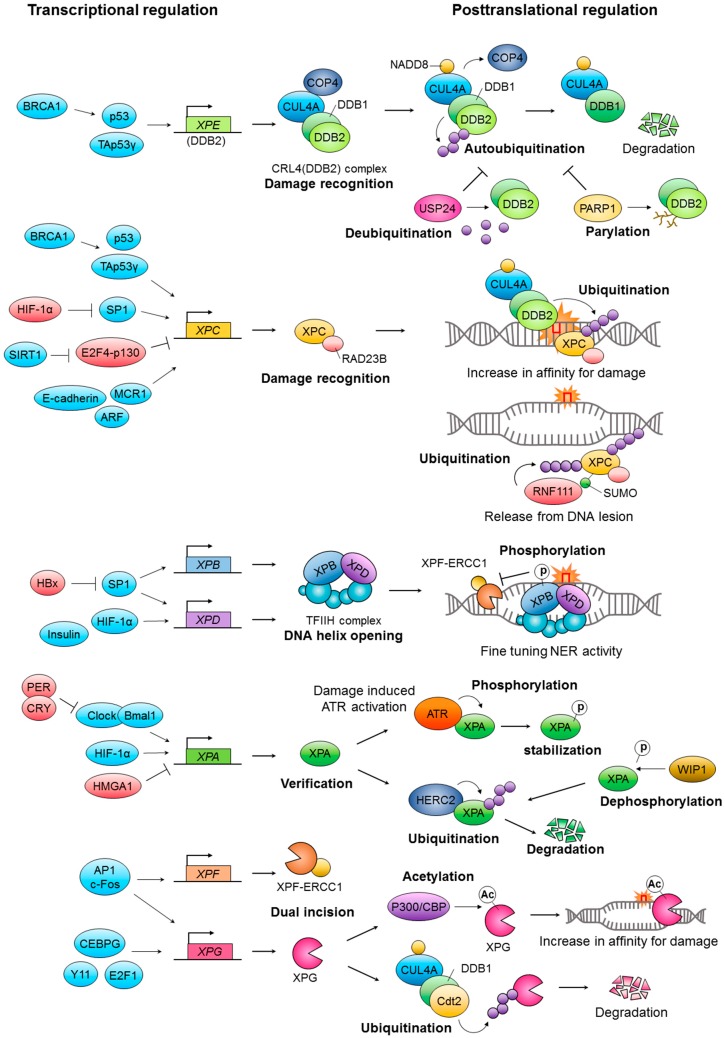
Schematic illustration for transcriptional and posttranslational modifications of core nucleotide excision repair (NER) factors. See text for detailed description.

Nucleotide excision repair (NER) is the only DNA repair system able to remove UV-induced photolesions in placental mammals [8,9]. NER also can repair other bulky DNA adducts, reactive oxygen species-induced base modifications, and intrastrand crosslinks [10]. The two distinct damage recognition subpathways that comprise NER are global genome NER (GG-NER) and transcription-coupled repair (TC-NER). The GG-NER subpathway is activated by the DNA damage sensor protein xeroderma pigmentosum complementation group C (XPC), whereas the TC-NER subpathway is activated by the stalling of RNA polymerase II (RNAPII) during transcription [11]. Hereditary mutations in NER-associated genes are associated with disorders that are characterized by UV sensitivity and cancer predisposition, such as xeroderma pigmentosum, Cockayne syndrome, and trichothiodystrophy [12,13]. Therefore, an understanding of the precise mechanisms underlying NER is needed to develop clinical interventions to counteract skin aging and cancer. In this review we highlight recent findings on the regulation of NER activity at the transcriptional and posttranslational levels (Figure 1).

## 2. Transcriptional Regulation of Nucleotide Excision Repair (NER)

### Transcriptional Regulation of Core NER Factors

Successful NER requires at least seven core NER factors (i.e., xeroderma pigmentosum (XP) proteins XPA–XPG) [14]. In Table 1, we summarized factors implicated in the transcriptional regulation of core NER factors.

The XPA protein is a rate-limiting factor for the entire NER process, and its expression level determines NER capacity [36]. XPA is required for damage verification in both GG-NER and TC-NER subpathways, triggering dual incision of the lesion by recruiting endonucleases XPF and XPG [37,38]. The transcription of XPA is controlled by a molecular circadian clock composed of a Clock-Bmal1 transcriptional activator and Cry-Per transcriptional repressor [39]. The daily oscillation of circadian clock activity results in the rhythmic expression of XPA in most differentiated mammalian tissues, including the brain and liver [15,16]. However, mice lacking clock activity due to the genetic knock-out of Cry show no rhythmic XPA transcription and thus no daily oscillation of NER activity in the mouse liver tissue [16]. Other factors regulating XPA expression include high mobility group protein A1 (HMGA1) and hypoxia-inducible factor-1α (HIF-1α). The overexpression of HMGA1 in MCF-7 breast cancer cells downregulates XPA transcription and thus increases UV sensitivity [17,40], whereas HIF-1α binding to the hypoxia response element (HRE) in the *XPA* promoter upregulates XPA expression approximately five-fold, implying that targeting HIF-1α may improve chemo-efficacy [18].

XPB (also known as excision repair cross-complementation group 3, ERCC3) and XPD (ERCC2) are components of the transcription factor IIH (TFIIH) complex. These proteins function as ATP-dependent DNA helicases, opening DNA strands around the site of damage [41]. Expression of XPB is regulated by specificity protein 1 (Sp1), which binds to the *XPB* promoter and activates transcription [19,42]. Hepatitis B virus x (HBx) protein can bind to and inhibit Sp1 activity, thereby downregulating the expression of XPB [20,43]. The expression of XPD is regulated by HIF-1α, which binds to seven overlapping HRE regions in the *XPD* promoter [26], and by the insulin-dependent signaling pathway [31]. A long-term exposure to high glucose concentrations (>10 mM) induces a downregulation of the insulin-dependent increase in XPD mRNA levels, suggesting that glucose and insulin are important regulators of XPD transcription, and prolonged exposure to high levels of glucose may impair the insulin-dependent regulation of DNA repair.

The damage sensor XPC recognizes distortions in the DNA helix [44,45], and XPE (also known as DNA damage-binding protein 2 (DDB2)) recognizes and binds to UV-induced CPD, facilitating subsequent XPC binding [46]. After UV irradiation, the expression of XPC and XPE is increased in a p53-dependent manner. The *XPC* promoter contains a putative p53 response element that interacts with p53 in vitro [21]. Similarly, the *XPE* promoter contains a p53 binding site [32]. The p53-dependent upregulation of XPC and XPE expression in malignant melanoma correlates with enhanced NER activity and consequently with chemoresistance [22]. Transactivation isoform of p63 gamma (TAp63γ) is a p53 homolog that transcriptionally regulates p53 target genes [47]. Overexpression of TAp63γ stimulates expression of XPC and XPE at both mRNA and protein levels, further enhancing NER activity upon UV damage [23]. Breast cancer 1 (BRCA1), a key factor in DNA double-strand break repair, can enhance expression of XPC and XPE independent of p53 [24]. Defects in the NER pathway in BRCA1-associated breast cancers may be causal in tumor development. In addition, Sp1 binds to the *XPC* promoter to increase its expression after UV irradiation [25]. The Sp1-binding sequence overlaps the HRE sequence in the *XPC* promoter; thus HIF-1α competes with Sp1 at the same binding site. Under normal conditions, HIF-1α can suppress Sp1 binding, but with exposure to UV radiation, downregulation of HIF-1α enables Sp1 binding to the *XPC* promoter, thereby increasing XPC expression [26]. Sirtuin 1 (SIRT1) stimulates XPC expression by blocking the nuclear localization of the transcriptional repressor E2F4-p130 [27]. Akt activation as a result of SIRT1 inhibition is critical for the nuclear accumulation of the E2F4-p130 repressor complex. SIRT1 also can interact with Rb (retinoblastoma protein), as well as its family members p103 and p130, and to deacetylate Rb. It is possible that SIRT1 also acts as a deacetylase for p130, and thus plays an important role in regulating the function of the E2F4-p130 repressor complex in XPC transcription. Alternatively, Akt may phosphorylate p130, and thus loss of SIRT1 may increase the acetylation of p130, thus increasing nuclear p130 levels [27]. Similarly, the alternative reading frame (ARF) tumor suppressor disrupts the interaction of the E2F4-p130 with the *XPC* promoter to increase XPC expression levels [28]. The cell adhesion molecule E-cadherin increases XPC expression by disrupting E2F4-p130 transcription repressor complexes. Conversely, loss of E-cadherin activates the transforming growth factor beta (TGF-β) pathway, which upregulates E2F4-p130 and decreases XPC expression and NER activity [29]. Activation of melanocortin 1 receptor (MC1R) is associated with stimulation of XPC expression as well as ataxia telangiectasia and Rad3-related (ATR)-mediated H2AX phosphorylation to protect skin cells from UV irradiation [30].

XPF (ERCC4) and XPG (ERCC5) are structure-specific endonucleases that facilitate dual incision of the damaged strand [48]. The mRNA levels of XPF and XPG decline after UV irradiation but are quickly restored by activation of the transcriptional activation of c-Fos/AP-1 [33], which binds to the AP-1 binding site in the *XPF* promoter to induce its expression, increasing NER activity [34]. Thus, in *c-Fos* deficient cells, both *XPF* and *XPG* mRNA production are significantly reduced, which leads to decline of XPF protein levels after UV irradiation. Even though the XPG mRNA level is also deceased, the XPG protein level is not changed after UV irradiation due to the intrinsic higher stability of the XPG protein [33]. The XPF protein is stabilized by ERCC1, suggesting that ERCC1 expression influences XPF levels [49]. Transcriptional activation of *ERCC1* is stimulated by oncogenic H-Ras, which subsequently triggers AP-1 transcriptional activity [50]. AP-1 is also transcriptionally upregulated by glioma-associated oncogene homolog 1 (Gli1); thus inhibition of Gli1 by small hairpin RNA downregulates AP-1, thereby decreasing ERCC1 and XPF expression [51]. The expression of XPG is upregulated by CCAAT/enhancer-binding protein gamma (CEBPG), whose expression is modulated by E2F1 and YY1 (Yin Yang 1) [35]. CEBPG belongs to a member of the CEBP family of transcription factors. The CEBP family proteins form homodimers or heterodimers to regulate CCAAT/enhancer element-mediated transcription. In this regard CEBPG also can form heterodimers with other CEBP proteins to transcriptionally activate *XPG* transcription.

## 3. Posttranslational Regulation of NER Factors

### 3.1. Ubiquitination and SUMOylation

Posttranslational modification is a fundamental mechanism in the regulation of protein functions. Recent data show that the activity of NER factors is also under control of posttranslational modifications. In Table 2, we summarized factors involved in the posttranslational regulation NER factors. Ubiquitination—i.e., attachment of ubiquitin to lysine residues on substrates—is important for regulating NER activity [52]. In the initiation of GG-NER, ubiquitination of XPC is mediated by the cullin-RING ubiquitin ligase (CRL) complex, which consists of damage-specific DNA binding protein 1 (DDB1), DDB2, regulator of cullins 1 (ROC1), and the cullin 4A (CUL4A) scaffold [53]. CRL4(DDB2) is inactivated by constitutive photomorphogenesis 9 (COP9) signalosome deneddylation activity in quiescent cells [54]. When DNA damage is detected by DDB2, this complex is activated by neural precursor cell expressed developmentally downregulated protein 8 (NEDD8), an ubiquitin-like protein involved in protein neddylation [55,56]. CRL4(DDB2) can ubiquitinate histones, XPC, and DDB2 itself [57,58,59,60]. The degradation of DDB2 is induced by Lys48-linked auto-ubiquitination via the 26S proteasome, and this degradation is prevented by the deubiquitinating enzyme ubiquitin-specific processing protease 24 (USP24) [60,61]. However, DDB2-mediated XPC ubiquitination does not result in XPC degradation, and the protein retains its DNA-binding activity [53,62]. The activity of p38 mitogen-activated protein kinase (MAPK) kinase is required for DDB2 ubiquitination and degradation and for the recruitment of XPC and TFIIH onto sites of UV damage [63]. DDB2 phosphorylation at serine moiety by p38 MAPK facilitates ubiquitination and degradation of DDB2, but the specific site of serine phosphorylation by p38 MAPK and whether this phosphorylation is indispensable for UV-induced DDB2 ubiquitination has not been confirmed yet. UV radiation resistance-associated gene (UVRAG) protein, which is also required for efficient XPC recruitment, promotes the assembly and activity of the CRL4(DDB2) complex [64]. During UV exposure, UVRAG localizes to photolesions and associates with DDB1 to promote the assembly and activity of the DDB2-DDB1-Cul4A-Roc1 ubiquitin ligase complex, leading to efficient XPC recruitment to the lesions. Disruption of UVRAG-DDB1 interaction attenuates ubiquitination and association of XPC with UV-induced photolesions.

XPC is additionally modified by RING finger protein 111/Arkadia (RNF111) by Lys63-linked poly-ubiquitination. This modification stimulates XPC release from the DNA lesion, which is essential for completion of NER [65]. RNF111 selectively ubiquitinates SUMOylated XPC [66,67]. The SUMOylation of lysine residues 81, 89, and 183 of XPC promotes NER [68], whereas SUMOylation of lysine 655 is required for UV-induced XPC degradation [69].

When RNAPII stalls at the site of DNA damage, Cockayne syndrome protein B (CSB) stably binds to RNAPII and recruits CSA [11]. CSA then forms a complex with CUL4, ROC1 and DDB1 that appears to regulate the ubiquitination of CSB [70]. The ubiquitin-binding domain of CSB appears to be crucial for efficient TC-NER [71]. The UV-sensitive syndrome A (UVSSA) protein recruits USP7 to the stalled RNAPII, which promotes the deubiquitination of CSB, thereby counteracting the CSA-dependent ubiquitination of CSB and stabilizing CSB by inhibiting its degradation [72]. CSB also undergoes SUMOylation after UV irradiation, which is critical for the recruitment of CSA [73]. Therefore, UVSSA and CSA, through different ubiquitin-mediated mechanisms, seem to have opposite roles in determining the fate of CSB.

As noted previously, XPA protein levels exhibit circadian oscillation. The control of XPA is accomplished by circadian clock-mediated transcriptional regulation and homologous to the E6-AP carboxyl terminus (HECT) domain and RCC1-like domain-containing protein 2 (HERC2)-mediated posttranslational regulation [16]. The HERC2-mediated ubiquitination of XPA leads to its degradation, which contributes to the high amplitude of XPA circadian oscillation [36]. After UV-induced DNA damage occurs, XPA recruits XPF-ERCC1 and XPG endonucleases to remove the damaged DNA strand. ERCC1 can be deubiquitinated by USP45, which promotes XPF-ERCC1 recruitment to sites of DNA damage [74]. Upon UV exposure, USP45 interacts with ERCC1–XPF but the USP45 mutant (Asp25Ala, Glu26Ala) that is catalytically active but that cannot bind to ERCC1 failed to deubiquitinate ERCC1. The major role of ERCC1 deubiquitination by USP45 has been speculated to enable ERCC1–XPF to gain access to DNA damage sites. After XPG cleaves strands on the 3′ side of the lesion, XPG elimination from the DNA is important for subsequent DNA repair. XPG is degraded in response to both UV and cisplatin treatments, and CRL4(Cdt2)-mediated XPG degradation facilitates the recruitment of DNA polymerase δ and DNA repair synthesis [75].

### 3.2. Phosphorylation, Acetylation, and PARylation

The DNA damage response involves the coordination of multiple cellular responses including DNA repair, cell cycle checkpoint control, DNA replication, and apoptosis [88]. The ATR protein kinase, which is the major regulator of the DNA damage response, phosphorylates proteins associated with cell cycle checkpoints and DNA repair. ATR phosphorylates XPA at serine 196, which is located in the DNA-binding domain [76]. A phospho-deficient XPA mutant (S196A) exhibits greater UV sensitivity compared with the wildtype, while the phospho-mimic form (S196D) shows tolerance to UV damage. In addition, the downregulation of ATR in the presence of UV damage results in the destabilization of XPA, and S196A expressed in XPA cells compromises NER activity [77]. UV-induced phosphorylation of XPA by ATR stimulates NER by inhibiting HERC2-mediated ubiquitination, which promotes XPA stabilization and chromatin retention [77]. ATR also regulates nuclear translocation of XPA through interaction with importin-α4 [89]. The binding of importin-α4 to XPA is dependent on UV-irradiation. Knockdown of ATR reduces the amount of XPA interacting with importin-α4. Nuclear-Dbf2-related kinase 1 (NDR1) is a novel XPA-interacting protein that indirectly modulates NER activity via the ATR pathway [90]. The NDR1-depleted cells display reduced activity of ATR during UV-induced DNA damage, hypothesizing that NDR1 functions as a transducer of DNA damage signals to ATR. Wild-type p53-induced phosphatase 1 (WIP1) dephosphorylates XPA; thus overexpression of WIP1 decreases NER activity [78]. WIP1-deficient mice show accelerated repair kinetics on the UV lesions and less apoptosis than their wild-type counterparts. In vitro phosphatase assays have identified XPA and XPC as two potential WIP1 targets, thus WIP1 may repress NER activity by dephosphorylating and inactivating XPA and XPC after repair.

E2F1 has recently been identified as a novel NER-regulating factor that accumulates at sites of UV-induced DNA damage [91]. ATR phosphorylates E2F1 at serine 31, which increases E2F1 protein stability and its recruitment to sites of damage [92]. E2F1 then recruits GCN5 acetyltransferase, which mediates histone acetylation required for efficient NER [93]. Phosphorylation sites of CSB have been explored using a proteomic analysis. Among multiple putative phosphorylation sites, serine 1461 has been found as a critical target for ATM and ATR-mediated phosphorylation, since this is inducible in response to ionizing radiation [94,95]. In addition, tyrosine 932 has been defined to be phosphorylated by the Abelson murine leukemia viral oncogene homolog 1 (c-Abl) kinase. Oxidative DNA damage such as hydrogen peroxide induces CSB phosphorylation at tyrosine 932 by c-Abl, thereby regulating the subcellular localization of CSB to facilitate repair activity [86]. The XPB subunit of TFIIH is phosphorylated at serine 751 of XPB in vivo [80]. XPB phosphorylation does not alter TFIIH helicase activity but inhibits 5′ incision induced by XPF-ERCC1, which is regarded as a critical point for fine-tuning the NER activity. Expression of a phosphomimetic XPB-S751E mutant is unable to compensate the NER defect of XP-B cells, whereas XPB-S751 dephosphorylation or expression of a phospho-deficient mutant (S751A) can restore NER activity.

It has been reported that NER activity can be decreased by XPA acetylation and increased by XPA deacetylation [79]. Deacetylation of XPA is required for its interaction with replication protein RPA32 [79]. A recent report demonstrated that a non-lethal dose of UV radiation induces SIRT1 expression, which promotes the binding of XPA to the damaged DNA, consequently fostering NER activity following lethal dose of cisplatin treatment, hence explaining chemoresistance mechanism of SIRT1-overexpressing cancer cells [96]. Ras association domain-containing protein 1 (RASSF1A) modulates XPA acetylation in a SIRT1-dependent manner [97]. In the presence of SIRT1 inhibitor, RASSF1A is no longer able to promote XPA deacetylation. XPG is acetylated by p300 acetyltransferase and its homolog CREB-binding protein (CBP) in vivo, which facilitates XPG accumulation at sites of DNA damage in a PCNA-p21 dependent manner [85,98]. Knockdown of both p300/CBP by RNAi or by chemical inhibition with curcumin significantly reduces XPG acetylation, and a concomitant accumulation of the protein at DNA damage sites is observed. The ability of p21 to bind PCNA is found to regulate the interaction between p300 and XPG [99]. In addition, AKT-mediated p300 phosphorylation at serine 1834 increases histone acetylation, which allows NER factors access to the damaged DNA [100].

Poly(ADP-ribose) polymerases (PARPs) catalyze adenosine diphosphate (ADP)-ribosylation using nicotinamide adenine dinucleotide as a substrate, transferring ADP-ribose to an acceptor protein [101]. Inhibiting PARP enzymatic activity is an effective strategy to target cancer and decrease chemotherapy toxicity due to compromised DNA repair [102,103]. DNA damage induces PARP1 activity, which is required for UV-induced DNA repair [104]. Results of an in vitro PARP assay show that DDB2 is a target for PARP1-mediated PARylation, which promotes interaction between PARP1 and XPC and increases localization of XPC to DNA lesions [84]. PARP1-mediated PARylation stabilizes DDB2 by inhibiting its ubiquitination and regulates the recruitment of the chromatin remodeling enzyme amplified in liver cancer protein 1 (ALC1) [83]. XPA has a high binding affinity for PAR chains, and the PAR-binding motif overlaps the DDB and TFIIF interaction domains of XPA [105,106]. PARP1 regulates the association of XPA to damaged chromatin, and XPA in turn stimulates PARP1 enzymatic activity [107]. In addition, PARP1 can PARylate both N- and C-terminals of CSB after oxidative DNA damage, which inhibits the ATP hydrolysis activity of CSB [87]. It will be intriguing to investigate how PARylation affects the interplay of the NER factors at DNA damage sites and to determine whether PARylation is a critical regulatory factor for coordinating the repair mechanisms.

## 4. Concluding Remark

Recent studies show that hotspot somatic mutations in cancer genomes include active gene promoters where the binding of transcription factors impairs NER activity [108,109]. These findings emphasize the critical importance of NER in maintaining genome stability. Understanding the transcriptional and posttranslational regulation of NER factors may enable the manipulation of NER activity to protect skin from UV-mediated damage and enhance the efficacy of chemotherapeutic agents. For cancer patients, pharmacological inhibition of NER is expected to improve chemosensitivity but nontoxic NER inhibitors are rare. This highly important issue needs more intense exploration.

## Figures and Tables

**Table 1:** A summary of transcriptional regulation of core nucleotide excision repair (NER) factors.

XP ^1^	Function in NER	Transcriptional Regulation		
		Factor	Regulation	Reference
XPA	Damage verification	Cry1	Down	[15,16]
		HMGA1	Down	[17]
		HIF-1α	Up	[18]
XPB	ATP-dependent helicase	Sp1	Up	[19]
		HBx	Down	[20]
XPC	Damage recognition	p53	Up	[21,22]
		TAp63γ	Up	[23]
		BRCA1	UP	[24]
		Sp1	Up	[25,26]
		HIF-1α	Down	[26]
		E2F4-p130	Down	[27,28]
		SIRT1	UP	[27]
		ARF	UP	[28]
		E-cadherin	Up	[29]
		MC1R	Up	[30]
XPD	ATP-dependent helicase	HBx	Down	[20]
		HIF-1α	Up	[26]
		Insulin	Up	[31]
XPE	Damage recognition	p53	Up	[22,32]
		TAp63γ	Up	[23]
		BRCA1	Up	[24]
XPF	5′ incision endonuclease	c-Fos, AP-1	Up	[33,34]
XPG	3′ incision endonuclease	c-Fos, AP-1	Up	[33]
		CEBPG, E2F1, YY1	UP	[35]

^1^ XP: xeroderma pigmentosum.

**Table 2:** A summary of posttranslational modification of NER factors.

XP	Modification	Factor	Residue	Effect	Ref.
XPA	Phosphorylation	ATR	Ser196	Stabilization	[76,77]
	Dephosphorylation	WIP1	Ser196	Inactivation	[78]
	Ubiquitination	HERC2	-	Degradation	[16,36]
	Deacetylation	SIRT1	Lys63, Lys67	Stabilization, Interaction with RPA32	[79]
XPB	Phosphorylation	-	Ser751	Inhibition of 5′ incision by XPF/ERCC1	[80]
XPC	Ubiquitination	CRL4(DDB2)	-	Affinity for DNA lesions	[53,59]
	Ubiquitination	RNF111	-	Release from DNA lesions	[66]
	SUMOylation	SUMO2	-	RNF111 induced ubiquitination	[65,66]
	SUMOylation	SUMO1	Lys81, Lys89, Lys183	Damage recognition	[67,68]
	SUMOylation	-	Lys655	Degradation	[69]
	Dephosphorylation	WIP1	Ser892	Inactivation	[78]
XPD	ISGylation	HERC5	-	Not investigated	[81]
	Ubiquitination	-	Lys 701	Possible ubiquitination site	[82]
XPE (DDB2)	Ubiquitination	CRL4(DDB2)	Lys5, Lys11, Lys35, Lys40, Lys151	Autoubiquitination, Degradation	[54,60]
	Deubiquitination	USP24	-	Stabilization	[61]
	PARylation	PARP1	-	Stabilization	[83,84]
XPG	Ubiquitination	CRL4(Cdt2)		Degradation	[75]
	Acetylation	p300/CBP		Affinity for DNA lesions	[85]
CSB	Ubiquitination	-	-	Degradation	[70]
	Deubiquitination	USP7	-	Stabilization	[72]
	SUMOylation	SUMO2/3	Lys205	Efficient TC-NER	[73]
	Phosphorylation	c-Abl	Tyr932	Nuclear localization	[86]
	PARylation	PARP1	-	Repair for oxidative DNA damage	[87]
ERCC1	Deubiquitination	USP45	-	Access to damage site	[74]

## Abbreviations

6-4PP	Pyrimidine-pyrimidone (6-4) photoproduct
ADP	Adenosine diphosphate
ALC1	Amplified in liver cancer protein 1
AP-1	Activator protein 1 (also known as c-Jun)
ARF	Alternative reading frame
ATR	Ataxia-telangiectasia mutated and RAD3-related protein kinase
BRCA1	Breast cancer 1
c-Abl	Abelson murine leukemia viral oncogene homolog 1
c-Fos	Proto-oncogene c-Fos
CBP	CREB-binding protein
CEBPG	CCAAT/enhancer-binding protein gamma
COP9	Constitutive photomorphogenesis 9
CPD	Cyclobutane pyrimidine dimer
CRL4	Cullin 4A (CUL4A)–regulator of cullins 1 (ROC1) E3 ubiquitin ligase
Cry1	Cryptochrome 1
CSB	Cockayne syndrome protein B
CUL4A	Cullin 4A
DDB1	DNA damage binding protein 1
ERCC1	Excision repair cross-complementation group 1
GG-NER	Global genome repair
Gli1	Glioma-associated oncogene homolog 1
HBx	Hepatitis B virus x protein
HECT	Homologous to the E6-AP carboxyl terminus
HERC2	HECT domain and RCC1-like domain-containing protein 2
hHR23B	Human UV excision repair protein RAD23 homolog B
HIF-1α	Hypoxia-inducible factor-1α
HMGA1	High-mobility group A 1
HRE	Hypoxia response element
ISGylation	Protein conjugation by interferon-stimulated gene 15
MAPK	Mitogen-activated protein kinase
MC1R	Melanocortin 1 receptor
NAD	Nicotinamide adenine dinucleotide
NDR1	Nuclear-Dbf2-related family of Ser/Thr kinases 1
NEDD8	Neural precursor cell expressed developmentally down-regulated protein 8
NER	Nucleotide excision repair
PARP	Poly(ADP ribose) polymerases
PARylation	Poly(ADP-ribosyl)ation
RASSF1A	Ras association domain-containing protein 1
RLD	RCC1 like domain
RNAPII	RNA polymerase II
RNF111	RING finger protein 111
ROC1	Regulator of cullins 1
SIRT1	Sirtuin 1
Sp1	Specificity protein 1
SUMO	Small ubiquitin-related modifier
TAp63γ	Transactivation isoforms of p63γ
TC-NER	Transcription-coupled repair
TGF-β	Transforming growth factor-β
UBD	Ubiquitin-binding domain
USP	Ubiquitin-specific processing protease
UV	Ultraviolet
UVRAG	UV radiation resistance-associated gene protein
UVSSA	UV-sensitive syndrome A
WIP1	Wild-type p53-induced phosphatase 1
XPA	Xeroderma pigmentosum complementation group A
XPB	Xeroderma pigmentosum complementation group B (also known as ERCC3)
XPC	Xeroderma pigmentosum complementation group C
XPD	Xeroderma pigmentosum complementation group D (also known as ERCC2)
XPE	Xeroderma pigmentosum complementation group E (also known as DDB2)
XPF	Xeroderma pigmentosum complementation group F (also known as ERCC4)
XPG	Xeroderma pigmentosum complementation group G (also known as ERCC5)
YY1	Yin Yang 1
